# Solid Surface Vitrification Is Better than Slow Freezing for the Long-Term Preservation of Testicular Fragments from Prepubertal Collared Peccaries (*Pecari tajacu* Linnaeus, 1758)

**DOI:** 10.3390/ani15101488

**Published:** 2025-05-20

**Authors:** Andréia M. Silva, Ana G. Pereira, Gabriel S. C. Bezerra, Yuri G. Matos, Luana G. P. Bezerra, Alexsandra F. Pereira, Moacir F. Oliveira, Pierre Comizzoli, Alexandre R. Silva

**Affiliations:** 1 Laboratory on Animal Germplasm Conservation, Federal University of Semiarid Region—UFERSA, Mossoró 59625-900, RN, Brazil; andreia.m.silva@hotmail.com (A.M.S.); anagloriavet@gmail.com (A.G.P.); gabriel.bezerra@alunos.ufersa.edu.br (G.S.C.B.); yurigmatos@gmail.com (Y.G.M.); luana_grasielly@yahoo.com.br (L.G.P.B.); 2 Laboratory on Animal Biotechnology, Federal University of Semiarid Region—UFERSA, Mossoró 59625-900, RN, Brazil; alexsandra.pereira@ufersa.edu.br; 3 Laboratory on Animal Morphology, Federal University of Semiarid Region—UFERSA, Mossoró 59625-900, RN, Brazil; moacir@ufersa.edu.br; 4 Smithsonian Conservation Biology Institute, National Zoological Park, Veterinary Hospital, Washington, DC 20008, USA; comizzolip@si.edu

**Keywords:** testicular fragments, wild animal, cryopreservation, *Tayassu tajacu*

## Abstract

The cryopreservation of male gonadal tissue is critical to conserve genetic material and use it later via assisted reproduction. This study aimed to evaluate cryopreservation methods (slow freezing, SF; solid surface vitrification, SSV) as well as the optimal concentrations of intracellular cryoprotectants during the SSV of testicular tissue from prepubertal collared peccaries. Five pairs of testes were dissected on different days into small fragments (3 mm^3^) and allocated to a non-cryopreserved, a control group or one of three treatment groups: SF; SSV 3 M (1.5 M dimethyl sulfoxide [DMSO] plus 1.5 M ethylene glycol [EG]); or SSV 6 M (3 M DMSO plus 3 M EG). After one week of storage in liquid nitrogen, tissue samples were warmed and evaluated in terms of histology, viability, proliferative capacity potential, and DNA integrity. The scores for histological integrity and cellular damage for SF (2.08 ± 0.05 and 2.33 ± 0.07, respectively) were similar to the results found in SSV 6 M (1.93 ± 0.04 and 2.30 ± 0.07; *p* > 0.05). However, these scores were better when compared to SSV 3 M (1.87 ± 0.05 and 2.08 ± 0.06; *p* < 0.05). The percentage of cellular viability was around 57% after all preservation treatments (*p* > 0.05), which was lower than in the control group (88.8 ± 1.9%; *p* < 0.05). The SSV 6 M treatment was better than the other treatments regarding the proliferative capacity potential of spermatogonia cells (3.52 ± 0.03) (*p* < 0.05), although it was lower than in the control group (4.00 ± 0.12) (*p* < 0.05). Additionally, SSV 6 M led to the same DNA integrity (97.0 ± 0.7%) as in the control group (99.4 ± 0.3%). These collective findings suggest that the combination of SSV with 6 M cryoprotectants is the most efficient for the cryopreservation of testes from prepubertal collared peccaries.

## Simple Summary

Cryobanking is an essential tool in understanding and sustaining biodiversity. Among the multiple types of biological samples that can be preserved from wild animal species, testicular samples from healthy individuals offer additional possibilities to produce offspring, even after death. Testicular fragments can be cryopreserved using slow freezing or vitrification, but protocols vary according to the age or reproductive status of the donor. The present study demonstrated that solid surface vitrification was better than slow freezing for the preservation of testicular fragments from prepubertal collared peccaries. Specifically, a 6 M combination of dimethyl sulfoxide and ethylene glycol in the vitrification solution was the most efficient.

## 1. Introduction

Currently, the main drivers of global biodiversity decline are mainly associated with the degradation and destruction of ecosystems and habitats, as well as the direct overexploitation of species and climate change [1]. Due to the high rate of biodiversity loss, habitat protection and ex situ breeding combined with reintroduction programs might be insufficient to stop or even significantly slow down this process [2]. Therefore, the use of assisted reproduction technologies in association with the creation of biobanks has emerged as a critical option to protect endangered wild animal species [3]. Among the different germplasms that can then be stored in biobanks, testicular tissue opens up numerous opportunities, including the subsequent culture of spermatogonia to produce a large amount of mature gametes from a given individual of considerable genetic value [4,5].

Some attempts to improve testicular preservation protocols have been made for wild animal species, especially using the adult collared peccary (*Pecari tajacu* Linnaeus, 1758) [6,7]. This is an interesting model given that, despite being a species that is globally considered to be of the least concern [8], its population is in decline in several South American biomes, such as the Atlantic Forest and the Caatinga [9]. One of the major limitations is that former studies in peccaries have focused on the preservation of testicular tissue from adult individuals. However, research on some domestic species has shown that the preservation of male gonads is equally important in prepubertal individuals but requires adapted protocols [10,11]. Young individuals have not yet initiated the spermatogenesis process and, therefore, have a more homogeneous cellular population and no seminiferous tubule lumen in their gonads [10,12]. Importantly, the proper preservation of tissue from prepubertal animals offers the possibility to produce mature gametes at a later stage, either by xenografing or in vitro culture [13].

Different cryopreservation methods have been explored for male gonadal tissue, such as slow freezing (SF) and vitrification. SF is the most well-known and commonly used approach. Despite the advantage of using low and less toxic concentrations of cryoprotectants, the prevention of ice crystal formation in tissue is more challenging [14]. On the other hand, ultra-rapid freezing or vitrification involves the use of high concentrations of cryoprotectants, which transform into glass, without ice crystallization [15]. For testicular fragments from adult collared peccaries, the use of SF has proven to be more effective than solid surface vitrification (SSV) [7]. However, there are no data about the optimal protocols in prepubertal animals.

Both cryopreservation methods mentioned above require appropriate concentrations of cryoprotectants based on the species and the age of the individual. In the adult collared peccary, for instance, previous studies on testicular tissue suggested the use of 1.5 M (0.75 M DMSO and 0.75 M EG) for SF, while 3 M (1.5 M DMSO and 1.5 M EG) was recommended for SSV [7]. On the other hand, higher cryoprotectant concentrations of up to 4.7 M (2.1 M DMSO and 2.6 M EG) were indicated for the solid surface vitrification of testicular tissue from prepubertal pigs [16], as well as 5.6 M (2.8 M DMSO and 2.8 M EG) for prepubertal cats [17] and dogs [18].

The present study aimed to compare the methods of SF and SSV (using two different concentrations of cryoprotectants) in the preservation of the tissue morphology, viability, proliferative capacity potential, and DNA integrity in the testicular tissue of prepubertal collared peccaries. These animals came from groups that were not genetically related to each other.

## 2. Materials and Methods

### 2.1. Animal Ethics

All experimental protocols were approved by the Animal Ethics Committee of the Federal Rural University of the Semi-Arid Region (protocol no. 23091.005557/2017-17; opinion no. 04/2017 from 10 August 2017) and the Chico Mendes Institute for Biodiversity Conservation (no. 37329 from 15 July 2023). The prepubertal males were obtained from the Wild Animal Multiplication Center of our own institution, UFERSA (Mossoró, RN, Brazil).

### 2.2. Testis Collection and Experimental Design

Five prepubertal males of 3–6 months of age were used. Testes from one animal were collected on different days (one testis pair represented one replicate). After slaughter, the pairs of testes were washed in saline solution (NaCl 0.9%) and transported to the laboratory in an isothermal box (28 °C) for a maximum of 30 min.

**Figure 1:**
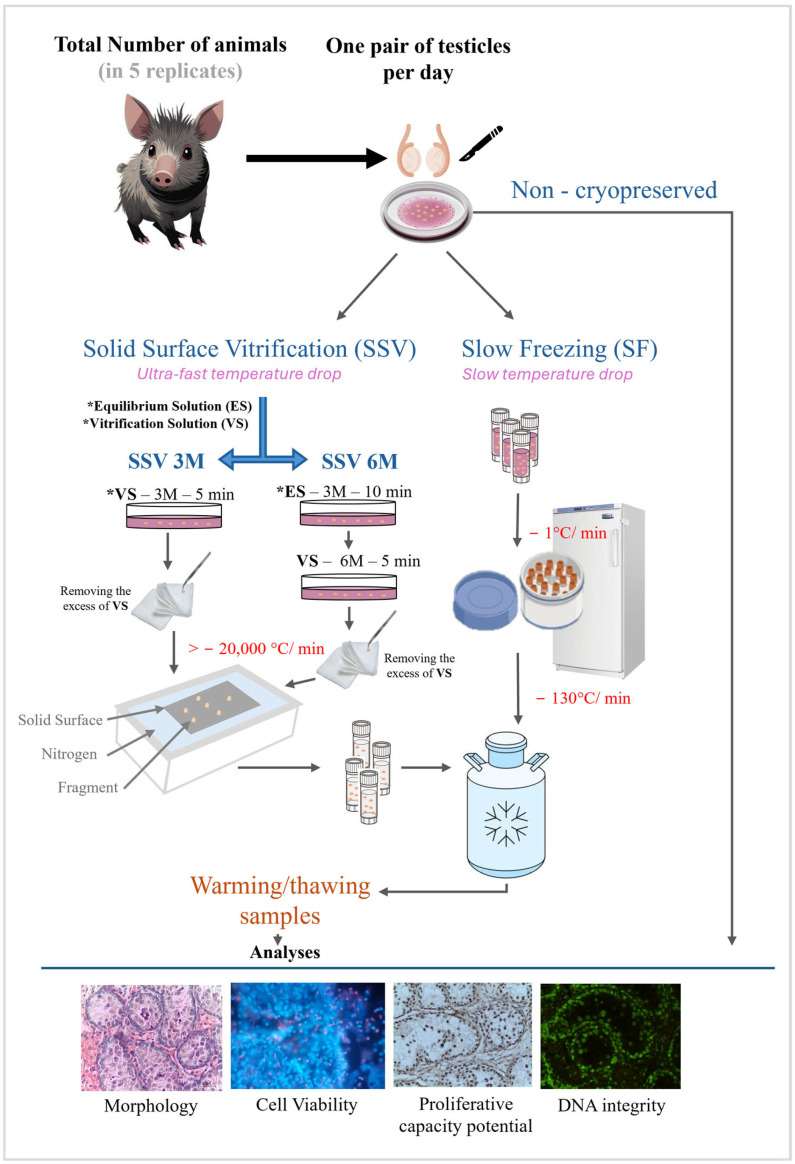
Experimental design.

In the laboratory, testes were isolated from surrounding tissue and washed with Minimum Essential Medium (MEM). Testicular tissue from a given male then was dissected into 46 fragments measuring 3.0 mm^3^ (3 × 1 × 1 mm), which were randomly allocated to non-cryopreservation (control group) or one of three cryopreservation treatments. All treatments included exposure to a combination of DMSO and EG (see below), followed by the combination of SF with 1.5 M of cryoprotectant or SSV with 3.0 M or 6.0 M of cryoprotectant (Figure 1). In each treatment group, two fragments were used for each of the following evaluations: histology, cell viability, cell proliferative capacity potential, and DNA integrity (Figure 1).

### 2.3. Cryopreservation and Thawing/Warming

The SF solution consisted of MEM supplemented with 0.25 M sucrose, 10% fetal bovine serum (FBS), 0.75 M DMSO, and 0.75 M EG [7]. For the SF procedure, 2.0 mL cryovials (Fisher Scientific, Pittsburgh, PA, USA) each contained 12 tissue pieces immersed in 2.0 mL of the SF solution at 24 °C [7]. The cryovials were then placed in a Nalgene freezing container (Mr. Frosty^®^, Thermo Fisher Scientific, Wilmington, NC, USA) filled with isopropyl alcohol at 25 °C. The Nalgene container was then transferred to a −80 °C freezer overnight (cooling rate −1 °C/min). Afterward, samples were moved to liquid nitrogen containers for one week of storage (cooling rate ~ −130 °C/min) [7,19].

The vitrification solution was composed of MEM supplemented with 0.25 M sucrose, 10% FBS, 3 M (1.5 M DMSO + 1.5 M EG), or 6.0 M (3.0 M DMSO + 3.0 M EG). Vitrification was performed using two different vitrification solutions, 3 M and 6 M, at 25 °C. In both treatments, fragments were exposed for 5 min to a vitrification solution before SSV. However, in the 6 M treatment, fragments were exposed to the 3 M solution for 10 min before being exposed to the vitrification solution. After removing the excess solution with an aseptic absorbent filter, the fragments were placed for 30 s on an aluminum sheet in contact with liquid nitrogen and then transferred in cryotubes for one week of storage in liquid nitrogen (cooling rate > −20,000 °C/min) [7].

For thawing/warming, cryovials were placed on a bench for 1 min at 25 °C, followed by immersion in a 37 °C water bath. All fragments were then washed three times for 5 min each in MEM supplemented with 10% FBS, followed by washing with decreasing concentrations of sucrose (0.50 M, 0.25 M, 0 M sucrose) to remove the cryoprotectants [17,20].

### 2.4. Testicular Cell Morphology Analysis

**Figure 2:**
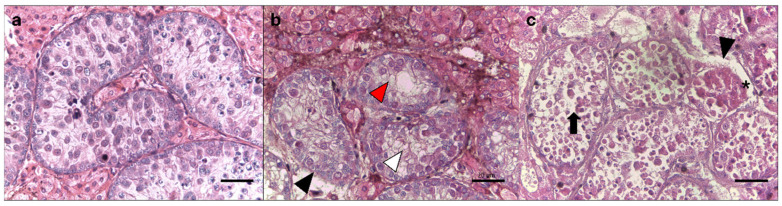
Morphological parameters of seminiferous tubules in testes from collared peccaries. (**a**) Normal tissue morphology (score 3 for all parameters). (**b**) Partly ruptured from basal membrane (score 2—black head arrow), >50% cells without swelling (score 2; red head arrow), >50% cells without swelling (score 3; white head arrow). (**c**) Loss of tubular morphology (score 1), mostly ruptured from the basal membrane (score 1; black head arrow), with all cell types present, albeit with a slightly disordered structure and loss of tubular morphology (score 2—black arrow) and shrinkage from the basal membrane (score 2—asterisk). Scale bar, 20 µm.

For histological assessment, fragments from both the control and cryopreserved groups were fixed in Bouin’s solution for 24 h, sectioned at 5.0 µm, stained with hematoxylin–eosin, and examined under a brightfield microscope (Olympus CX 31 RBSFA, Tokyo, Japan). Morphological parameters included the tubular structure, ruptures of the basement membrane, swelling, tubular cell loss, separation of the basal membrane, and vacuolization (Table 1, Figure 2) [6]. Testicular tissue samples were then classified, with scores ranging from 0 to 3, where a score of 0 indicated a poor testicular morphology and a score of 3 represented morphologically normal tissue (Table 1). In each control and treatment group, 30 seminiferous tubules were evaluated.

### 2.5. Testicular Cell Viability

Testicular cells from non-cryopreserved fragments were first dissociated using enzymatic digestion [16]. Briefly, fragments were exposed to 0.2% collagenase type IV in MEM at 37 °C for 10 min under slow stirring, followed by the addition of an equal volume of FBS to stop the enzymatic reaction [16]. For cryopreserved samples, fragments did not require enzymatic digestion due to the disaggregation caused by cryopreservation (fragments were flattened between a slide and coverslips). Ten µL of isolated cells was incubated in a solution composed of 3 µL Hoechst 33342 (40 µg/mL in phosphate-buffered saline—PBS) and 3 µL of propidium iodide (0.5 mg/mL in PBS) for 10 min at 37 °C. Then, a total of 100 cells were counted and classified as non-viable or membranes without structural integrity (red fluorescence; propidium iodide) or viable with a membrane with structural integrity intact (blue fluorescence; Hoechst 33342).

### 2.6. Proliferative Capacity Potential of Testicular Cells

Tissue fragments from the control and cryopreserved groups were fixed in 4% paraformaldehyde solution for 12 h, embedded in paraffin blocks, and sectioned (5.0 µm thickness). The proliferative capacity was assessed by quantifying nucleolar organizer regions (NORs) in spermatogonia and Sertoli cells using the silver staining technique. Tissue sections mounted on slides were immersed in a silver solution (1 part of 2% gelatin in 1% aqueous formic acid and 2 parts of a 50% aqueous silver nitrate solution) in a dark room for 30 min. Next, the slides were washed in a 5% thiosulfate solution for 10 min. For each group, the NOR dots were counted within the nucleoli of spermatogonia and Sertoli cells across 10 randomly selected nuclei in 10 fields at 1000× magnification [21]. Cells were identified based on their localization and nuclear morphology [22].

### 2.7. DNA Integrity

The testicular tissue was fixed overnight in a 4% paraformaldehyde solution, embedded in paraffin, and sectioned into 5-µm-thick slices. Sections were then mounted on Starfrost^®^ slides (Knittel Glass, Braunschweig, Germany) and assessed using the In-Situ Cell Death Detection Kit (Roche, Basel, Switzerland), following the manufacturer’s protocol. Tissue sections were washed twice for 10 min in xylene and then gradually rehydrated through a series of ethanol dilutions (from 100% to 70%). Tissue sections were subsequently rinsed twice for 5 min in 0.05% Triton X-100 in PBS, permeabilized with 0.5% Triton X-100 in PBS for 30 min, and rinsed again for 5 min in 0.05% Triton X-100 in PBS.

The TUNEL reaction mixture was prepared by combining 5 µL of terminal deoxynucleotidyl transferase (TdT) enzyme solution with 45 µL of nucleotide polymer label solution. Tissue sections were incubated with 50 µL of this TUNEL mixture for 1 h at 37 °C in a humidified, dark environment. A negative control was created using only the label solution (without TdT), while a positive control involved a 10 min incubation period with 5 μL of recombinant DNase I (Sigma-Aldrich, Burlington, MA, USA) and 45 μL of 0.05% Triton X-100 in PBS to induce DNA strand breaks before labeling. Nuclei were counterstained with Hoechst 33342 (1:100, Sigma-Aldrich) for 10 min in a humidified chamber at room temperature. Slides were then mounted with 100 µL of Vectashield mounting medium (Vector Laboratories, Newark, CA, USA).

A total of 25 images per treatment group were captured with the SPOT Advanced software 5.0 using an Olympus BX41 epifluorescence microscope (Olympus Corporation, Tokyo, Japan). Images were analyzed using the ImageJ software (version 1.41). TUNEL-positive cells, stained with green fluorescence, were classified as damaged, while TUNEL-negative cells, stained with blue fluorescence, were considered normal [10].

### 2.8. Statistical Analysis

Values were expressed as the mean and SEM of the 5 replicates. They were then tested for normality and homoscedasticity using the Shapiro–Wilk and Levene tests, respectively (Stat View 5.0, SAS Institute, Inc., Cary, NC, USA). The effects of cryoprotectants on the testicular parameters were assessed by ANOVA, followed by Tukey’s test. Scores obtained in classical histology were subjected to the Mann–Whitney test for comparisons among the treatment groups. Differences were considered significant when *p* < 0.05.

## 3. Results

### 3.1. Histomorphology

**Figure 3:**
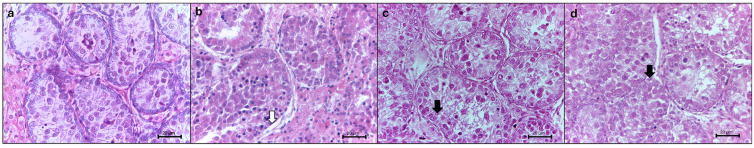
Morphological evaluation of testicular tissue of prepubertal collared peccaries using hematoxylin and eosin stains. (**a**) Control group, not cryopreserved; (**b**–**d**) groups cryopreserved by slow freezing (**b**) and solid surface vitrification using 3 M (SSV 3 M) (**c**) and 6 M (SSV 6 M) cryoprotectants (**d**). The white arrow indicates shrinkage from the basal membrane. Black arrowheads show cell swelling. The black arrowhead shows rupture from the basal membrane. Scale bar: 20 µm.

The histomorphological aspects of the fresh and frozen testicular tissue are shown in Table 1 and Figure 3. Overall, the SF and SSV 6 M groups exhibited similar outcomes based on all parameters (*p* > 0.05). Specifically, the seminiferous tubules’ basal membrane integrity was better preserved with SSV 6 M and SF than SSV 3 M. The same treatment also prevented cell swelling in comparison to SSV 3 M (*p* < 0.05). However, the SF group exhibited the most effective preservation of the tubular structure, while greater tubular cell loss was observed in SSV 6 M in comparison to SSV 3 M (*p* < 0.05) (Table 2).

### 3.2. Cell Viability

**Figure 4:**
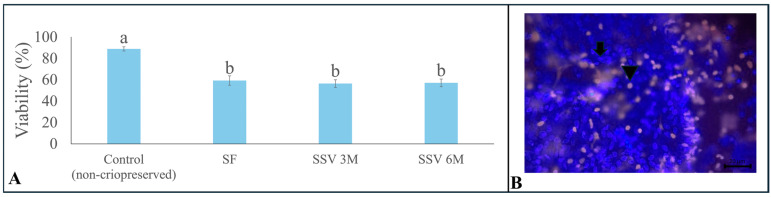
Percentages (means ± SEM) of viable cells in testicular tissue from prepubertal collared peccaries exposed to slow freezing (SF) and solid surface vitrification using 3 M (SSV 3 M) or 6 M (SSV 6 M) cryoprotectants (**A**). Representative micrograph of the evaluation of cell viability, in which the arrow shows the damaged cell (red fluorescence—probe: propidium iodide) and the arrowhead shows the intact cell (blue fluorescence—probe: Hoechst 33342) (**B**). Lowercase letters (“a, b”) above bars indicate significant differences among treatments according to Tukey test (*p* < 0.05). Scale bar: 20 µm.

Overall, all cryopreservation methods were able to maintain cell viability at around 57% after warming, which was lower than for the fresh control group (88.8 ± 1.9; *p* < 0.05). Moreover, there were no differences (*p* > 0.05) among the cryopreservation methods regarding testicular cell viability (Figure 4A).

### 3.3. Proliferative Capacity Potential

**Figure 5:**
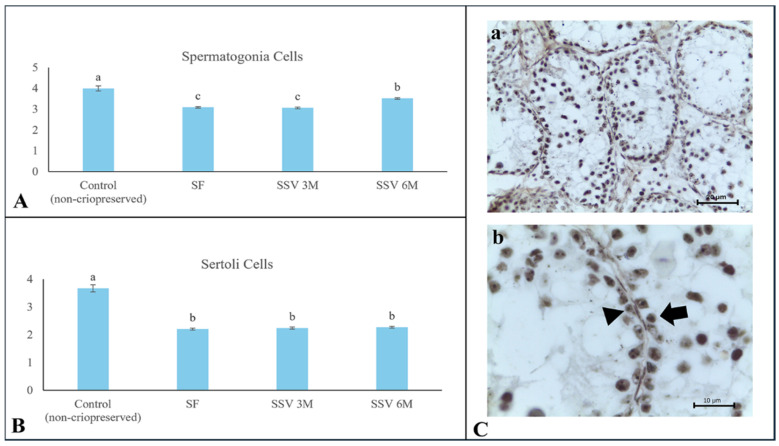
Proliferative capacity potential evaluated by quantification of nucleolar organizer regions (NORs) in testicular tissue from prepubertal collared peccaries after slow freezing (SF) or solid surface vitrification using 3 M (SSV 3 M) or 6 M (SSV 6 M) cryoprotectants. (**A**) Spermatogonia; (**B**) Sertoli cells; (**C**) NORs in testicular cells (black arrow and arrowhead). (**a**) Seminiferous tubule, scale bar 100 µm; (**b**) magnified seminiferous tubule area showing Sertoli cell (black head arrow) and spermatogonia (black arrow), scale bar 20 µm. Lowercase letters (“a, b, c”) above bars indicate significant differences among treatments according to Mann–Whitney test (*p* < 0.05).

The average values for the NORs calculated in the non-cryopreserved control group were 4.0 ± 0.1 for spermatogonia and 3.7 ± 0.1 for Sertoli cells. For both cell types, cryopreservation negatively affected the proliferative capacity potential, regardless of the freezing method (*p* < 0.05). Among these treatments, SSV 6 M provided the most effective preservation of the proliferative capacity potential for spermatogonia (3.5 ± 0.1 NORs; Figure 5).

### 3.4. DNA Integrity

**Figure 6:**
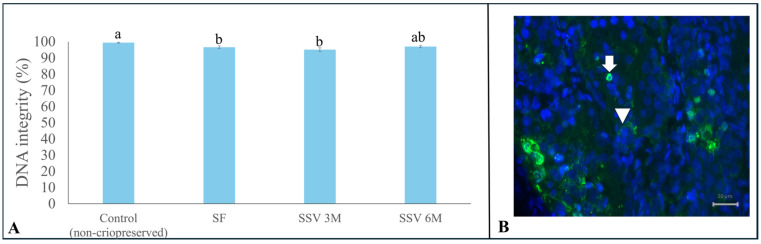
Percentages (means ± SEM) of cells with intact DNA in testicular tissue from prepubertal collared peccaries exposed to slow freezing (SF) and solid surface vitrification using 3 M (SSV 3 M) or 6 M (SSV 6 M) cryoprotectants (**A**). The arrow shows the damaged cell (green fluorescence) and the arrowhead shows the intact cell (**B**). Lowercase letters (“a, b”) above bars indicate significant differences among treatments according to Tukey test (*p* < 0.05). Scale bar: 20 µm.

Despite all cryopreservation protocols being similar regarding DNA integrity (*p* > 0.05) after testicular tissue thawing/warming (Figure 6), SSV 6 M (97.0 ± 0.1%) was the only one that provided values similar to the control group (99.4 ± 0.3%) (Figure 6).

## 4. Discussion

Our results demonstrate that SSV was more efficient than SF in preserving testicular tissue from prepubertal collared peccaries. DNA integrity was conserved only with SSV 6 M. Among these treatments, SSV 6 M also provided the most effective preservation of the proliferative capacity potential for spermatogonia. These findings differ from those of previous reports in adult individuals of the same species, in which SF showed better results than SSV [7]. This finding is consistent with the fact that vitrification has been largely used for testicular preservation in prepubertal individuals of murine [23] and feline species [24,25]. However, this does not mean that vitrification cannot be recommended for adult individuals, since the method has already proven to be efficient for mature testicular tissue from wild boars [26], deer, wolves, and black bears [27].

Regarding the testicular histomorphology, we verified that SSV 6 M and SF were similar in terms of preservation for all parameters. The combination of DMSO and EG at a concentration of 6 M during vitrification was necessary for the preservation of the morphology; however, 1.5 M could preserve these parameters during SF as well. In both methods, the combination of cryoprotectants minimized damage to the testicular tissue samples at the different cooling rates. The combined protection of the cryoprotectants used during freezing was essential, as DMSO can cause cellular dehydration and consequently reduce the possibility of ice crystal formation in cells during cryopreservation by inducing the formation of pores in the lipid membrane [28,29]. EG can permeate the cell membrane rapidly due to its low molecular weight, resulting in rapid action through preventing the formation of ice crystals via its ability to bind to water molecules [30,31].

Overall, SSV was even more efficient in preserving testicular tissue from prepubertal collared peccaries when using the highest concentration of cryoprotectants (6 M). It is likely that the concentration of 3 M of cryoprotectants was not sufficient to allow the preservation of the testicular tissue of prepubertal collared peccaries during the vitrification process. In fact, higher concentrations of cryoprotectants are generally required to reach a glassy state. This occurs in systems that are highly concentrated or that freeze very quickly, causing the viscosity to increase rapidly and preventing the molecules from organizing into a crystalline structure [32,33]. As cooling continues, the viscosity increases to the point at which molecular movement slows significantly, transforming the liquid into a glass. The final solid maintains the random molecular arrangement of the liquid but has the mechanical properties of a solid [32].

SSV 3 M provided the lowest scores for the tubular structure, tubular cell swelling, and tubular cell loss. Tubular cell loss could be related to failure in the preservation of the Sertoli cell cytoskeleton, which contributes to the collective organization of the seminiferous epithelium. Morphological studies have shown that it maintains shapes and forms and stabilizes the cell membrane at sites of cell–cell and cell–extracellular matrix contact. It consists of three major components, namely actin, intermediate filaments, and microtubules [34]. If the cytoskeletal structure and intercellular interactions are disrupted, this can lead to alterations in the tubular architecture and the subsequent loss of tubular cells. The cell–cell interaction reflects the fundamental level of physiological communication, triggering responses to internal or external environments and being essential for survival [35].

The use of SSV 3 M also increased the occurrence of tubular cell swelling. This alteration typically happens when cells lose their ability to regulate the movement of ions and water in and out of the cytosol in response to changes in osmotic pressure. It reflects the influx of sodium and water into the cell across the membrane, caused by malfunction or the insufficient capacity of the Na^+^/K^+^-ATPase pumps to exchange sodium for potassium at a rate that can maintain an appropriate water balance [36]. The high freezing rate with the low concentration of cryoprotectants did not allow enough time for the ionic channels to act and regulate the homotopic portion.

Regarding cell viability, all cryopreservation treatments were efficient (57% of viability), corroborating the results reported in prepubertal cats (~60%) [17] but lower than those observed in lambs (~80%) [37]. These similarities can be associated with age, since we used 3- to 6-month-old peccaries, as in the kitten study [17], while the lambs were 3- to 5-week-old neonates [37]. According to Amelkina et al. [10], who used a transcriptomic approach, it can be inferred that changes occur in the gene expression of testicular cells with age. Thus, the testes of juvenile cats, for example, are more resistant to damage caused by cryopreservation, and, as maturity approaches, their cells appear to become more sensitive [10,38]. Additionally, we highlighted that cell viability was directly related to the survival of cells, because the assay evaluated the cytoplasmic membrane integrity. This structure serves as a protective barrier, separating the internal components of the cell from the external environment. It provides a structure for the cell, thus being connected to the cytoskeleton, which helps to maintain the cell shape and organization, and is also involved in cell recognition and communication [39].

Vitrification with the highest cryoprotectant concentration (6 M) was the only treatment that effectively preserved the proliferative ability of the spermatogonia cells of prepubertal peccaries. Similar results were also obtained when a DMSO-EG combination was first used for the vitrification of testicular tissue from adult individuals in the same species [6]. This parameter was evaluated through the counting of NORs, which are DNA segments that transcribe ribosomal RNA, which is responsible for synthesizing all proteins, including those required for the formation of new cells [40,41]. The higher the number of NORs, the higher the possibility of spermatogonia proliferation and the production of sperm [21].

In addition, cellular proliferation is directly linked to DNA integrity. The TUNEL assay is based on the use of a terminal deoxynucleotidyl transferase (TdT) enzyme that attaches labeled dUTP to the 3′ ends of strand breaks. This method detects global DNA damage, including both single- and double-stranded DNA breaks [42]. When evaluating the treatments, SSV 6 M was also effective in preserving the DNA integrity, similarly to the fresh control; however, all treatments led to good DNA integrity (around 90%) for prepubertal peccary testicular cells. Interestingly, our results were higher than those found for adult peccaries (80%) [7] and similar to those found for prepubertal mice (up to 85%) [43]. It is likely that the prepubertal DNA is more resistant to cryopreservation due to fewer cellular variations and the absence of spermatocytes, in which the DNA is unpacked and can be more sensitive to degradation. According to Peris-Frau et al. [27], spermatogonia are in fact more resistant to cryodamage than other germ cells.

In general, both cryopreservation methods were simple and easy to perform in the laboratory; however, vitrification can be performed in the field since it does not require sophisticated devices or equipment, making it more practical, especially for wildlife [44,45]. It is also worth noting that further studies using in vitro or in vivo culture, such as xenotransplantation, are necessary to ensure the effectiveness of both methods in restoring spermatogenesis and producing viable spermatozoa [46]. The first steps of in vitro culture (IVC) for peccary testicular tissue were recently described by our team [47], providing a further possibility to test the efficiency of cryopreservation methods and indicating the possibility of using germplasm stored in biobanks.

## 5. Conclusions

In conclusion, we demonstrated that SSV using a 6 M solution of DMSO and EG was suitable for the preservation of testicular fragments from prepubertal collared peccaries. These results emphasize that, even for individuals of the same species, it is necessary to distinguish prepubertal from sexually mature males to establish an adequate testicular cryopreservation protocol.

## Figures and Tables

**Table 1:** Morphological parameters of seminiferous tubules in testes from collared peccaries.

Parameter	Score		
	#3	#2	#1
Tubular cell swelling	No swelling	>50% cells without swelling	>50% cells with swelling
Tubular cell loss	No cell loss	<75% cell types lost	>75% cell types lost
Rupture from basal membrane	No rupture	<50% partly ruptured	>50% mostly ruptured
Shrinkage from basal membrane	No shrinkage	<50% partly shrunken	>50% mostly shrunken
Tubular structure	Structure intact	All cell types present, although with slightly disordered structure	Random distribution of remaining cells

**Table 2:** Morphological evaluations (3—normal to 1—degenerated) of testicular tissue from prepubertal peccaries in non-cryopreserved (control) vs. cryopreserved groups (slow freezing, SF; solid surface vitrification with 3 M or 6 M, SSV 3 M, SSV 6 M).

	Control (Non-Cryopreserved)	SF	SSV 3 M	SSV 6 M
Tubular cell swelling	2.69 ± 0.05 ^a^	2.16 ± 0.06 ^b^	1.92 ± 0.06 ^c^	2.13 ± 0.04 ^b^
Tubular cell loss	2.81 ± 0.04 ^a^	2.47 ± 0.07 ^bc^	2.33 ± 0.07 ^c^	2.60 ± 0.06 ^b^
Rupture from basal membrane	2.74 ± 0.05 ^a^	2.33 ± 0.07 ^b^	2.08 ± 0.07 ^b^	2.30 ± 0.07 ^b^
Shrinkage from basal membrane	2.98 ± 0.02 ^a^	2.66 ± 0.07 ^b^	2.57 ± 0.07 ^b^	2.79 ± 0.04 ^b^
Tubular structure	2.80 ± 0.04 ^a^	2.08 ± 0.05 ^b^	1.87 ± 0.05 ^c^	1.93 ± 0.05 ^bc^

^a,b,c^ Different lowercase superscript letters indicate significant differences among treatments according to Mann–Whitney test (*p* < 0.05).

## Data Availability

The raw data supporting the conclusions of this article will be made available by the authors on request.
